# Investigating the Effect of Cold Soak Duration on Phenolic Extraction during Cabernet Sauvignon Fermentation

**DOI:** 10.3390/molecules20057974

**Published:** 2015-05-04

**Authors:** Siriwan Panprivech, Larry A. Lerno, Charles A. Brenneman, David E. Block, Anita Oberholster

**Affiliations:** 1 Faculty of Biotechnology, Assumption University, Bangkok 10240, Thailand; E-Mail: spanprivech@gmail.com; 2 Department of Viticulture and Enology, University of California, Davis, CA 95616-8749, USA; E-Mails: lalerno@ucdavis.edu (L.A.L.); cbrenneman@ucdavis.edu (C.A.B.); deblock@ucdavis.edu (D.E.B.)

**Keywords:** cold soak, phenolics, extraction, wine, Cabernet Sauvignon

## Abstract

The impact of increasing cold soak (CS) duration (0, 1, 4, 7, and 10 days at 10 °C) on the extraction of phenolic compounds during the CS period and primary fermentation as well as the final composition of Cabernet Sauvignon wine was investigated. The results showed that CS duration had no effect on hydroxycinnamate and flavonol extractions. Greater amounts of gallic acid, (+)-catechin, (−)-epicatechin, and total tannins were extracted with increasing CS duration, with differences maintained during bottle aging. Anthocyanin extraction and color density increased with longer periods of CS; however, by the end of primary fermentation, as well as three months’ bottle aging, there were no significant differences due to CS duration. The wines made with seven and 10 days of CS had higher seed tannin contributions and total tannin compared to the non-CS wine, which could potentially result in increased astringency.

## 1. Introduction

Phenolic compounds are important to red wine quality, as they are responsible for the color, mouthfeel, and ageability of wine. The phenolic composition of wine depends on the grapes used and also on the winemaking processes, as these will influence phenolic extraction into the must as well as subsequent reactions. The main phenolic compounds from a wine quality perspective are the anthocyanins, flavanols (including the oligomeric proanthocyanidins also referred to as condensed tannins), hydroxycinnamic acids, and flavonols. Phenolics are distributed throughout the grape berry, being found in the pulp, juice, skin, and seeds. Each component of the grape berry contains different classes of phenolic compounds, with each class contributing differently to the sensory properties of the wine. Proanthocyanidins and monomeric flavanols are found primarily in the grape skins and seeds and contribute to the bitterness and astringency of the wine. Anthocyanins are red pigments and the principal source of pigmentation in red wine. Anthocyanins are found in the skin of the grape berry for most *Vitis vinifera* varieties including Cabernet Sauvignon, as well as in the pulp of teinturier cultivars (e.g., Alicante Bouschet). Hydroxycinnamates are present throughout the grape berry, and react with anthocyanins as co-pigments, thereby stabilizing the color. Hydroxycinnamates are also strong antioxidants, and when oxidized can form brown pigments. The brown form has an effect on the color of white wine but only has a minor effect on the color of red wine. The flavonols are yellow pigments found in the cells of the grape skin. While less abundant than the other phenolics, the flavonols contribute to wine color as they are co-factors/pigments similar to hydroxycinnamates contributing to the color-enhancing phenomenon known as copigmentation [1,2,3,4].

Due to the significant influence of phenolic compounds on red wine quality, many winemaking processes have been developed to enhance the extraction of these compounds [1]. One such process is cold soak (CS), a period of prefermentative maceration lasting for several days (typically one to 10 days) in which the temperature of the must is kept low enough to prevent spontaneous fermentation (10–15 °C). It is claimed that CS favors the extraction of the more hydrophilic phenolic compounds, such as the anthocyanins, in the aqueous environment of the must as well as favoring skin tannin extraction over seed extraction [5]. Several studies examined the effect of CS on anthocyanin concentration in red wine with varying results. Gómez-Míguez *et al.* [6] showed that prefermentative cold maceration of Syrah grapes at 15 °C for seven days was successful at increasing the extraction of anthocyanins and other phenolic compounds, producing wines that were darker in color and less brown. De Santis and Frangipane [7] showed that a Merlot wine produced with CS at 8 °C for four days had a higher concentration of anthocyanins and volatile compounds than wines produced with traditional maceration. The CS technique promoted a high level of anthocyanin extraction in Cabernet Sauvignon wines when kept at 10 °C for seven days according to Gil-Muñoz *et al.* [8]. Busse-Valverde *et al.* [9] reported that CS (10 °C for 10 days) increased the seed proanthocyanidin concentration in Monastrell and Cabernet Sauvignon wines but had no effect on Syrah wines. Subsequently, they found that although CS also increased the extraction of anthocyanins in Monastrell wine it was not significant by the end of fermentation [10].

A study with Pinotage also found that CS at 10 °C improved the quality of the wine but did not produce a large difference in the phenolic compounds of the finished wine. CS decreased the concentration of acetate and ethyl esters with an increased skin contact time at 15 °C for two or four days prior to fermentation [11]. Reynolds *et al.* [12] found that Shiraz wine displayed an increase in anthocyanin extraction when CS (2 °C for 10 days) was combined with lower fermentation temperatures (15 and 20 °C), but not when combined with a high fermentation temperature (30 °C). Moreover, the effect of the time of CS on the evolution of phenolic compounds and color of Syrah wine was studied and it was found that 12 days’ cold maceration time resulted in wines with higher phenolic content in addition to more stable color with more red-bluish tonalities than shorter cold maceration time (8 days) and traditional maceration wines [13]. Peyrot des Gachons and Kennedy [14] showed that a CS of 4 and 10 days had no significant effect on the concentration of proanthocyanidins in the final Pinot noir wine.

Studies investigating CS have produced variable results, depending mainly on the duration of CS and grape variety. Previous studies have mostly focused on how CS affects the color and phenolic composition of the finished wine and did not evaluate phenolic evolution during the CS period or fermentation. This study investigated how CS duration affects phenolic extraction during the CS period and active fermentation as well as the final composition of Cabernet Sauvignon wine.

## 2. Results and Discussion

### 2.1. Chemical Composition of the Finished Wine 

The chemical composition of the wines made with different CS durations was determined at the time of bottling (Table 1). The results indicate that increasing the duration of CS had no effect on the basic chemical composition of the resulting wines (the percentage ethanol, pH, total acidity, acetic acid, malic acid, and residual sugar).

**Table 1:** Chemical composition of finished wines made with different CS durations.

Cold Soak Duration	% Ethanol	pH	Total Acidity (g/L)	Acetic Acid (g/L)	Malic Acid (mg/L)	Residual Sugar (g/L)
0 day	15.06 ± 0.10a	3.81 ± 0.02a	5.34 ± 0.03a	0.38 ± 0.01a	45 ± 4.0a	0.36 ± 0.01a
1 day	14.96 ± 0.08a	3.82 ± 0.03a	5.39 ± 0.03a	0.38 ± 0.01a	48 ± 1.0a	0.36 ± 0.02a
4 days	14.92 ± 0.10a	3.81 ± 0.05a	5.41 ± 0.14a	0.37 ± 0.02a	47 ± 2.0a	0.37 ± 0.01a
7 days	14.92 ± 0.02a	3.82 ± 0.03a	5.48 ± 0.03a	0.38 ± 0.02a	49 ± 3.5a	0.36 ± 0.02a
10 days	14.82 ± 0.22a	3.80 ± 0.04a	5.49 ± 0.17a	0.38 ± 0.01a	52 ± 0.6a	0.34 ± 0.01a

Notes: Means ± SD followed by same letter within the same column indicates no significant difference (*p* < 0.05, n = 3).

### 2.2. Chromatic Composition of the Must and Wines

**Figure 1:**
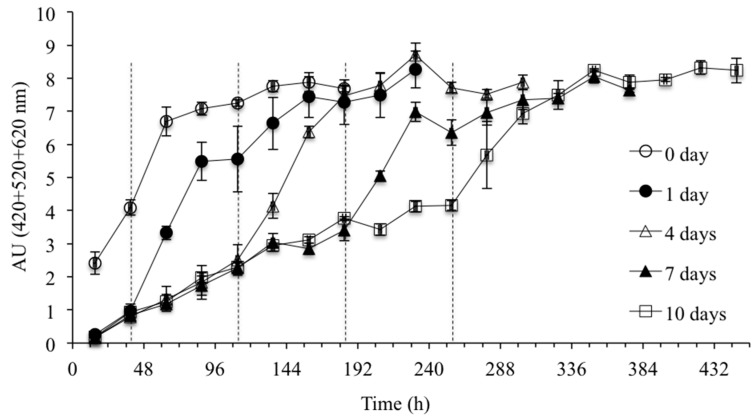
Color density evolution at different CS durations during CS and active fermentation as determined by UV-vis (n = 3). The end of CS for 1, 4, 7, and 10 days of CS treatment are marked as a dashed line at 39, 111, 183, and 255 h, respectively.

The evolution of color density during CS and active fermentation at different CS durations as determined by UV-vis are shown in Figure 1 (end of CS marked by a dashed line). The significant difference in color density between CS and non-CS (control) treatments is due to the fact that the first sampling point was at the start of fermentation for the non-CS treatment (15 h after inoculation) with simultaneous sampling of the different CS duration treatments as they reached CS maceration temperature (10 °C) at the same time point. This difference represents the impact of temperature on extraction as the control non-CS treatment was maintained at 25 °C whereas CS treatments (1 to 10 days) were cooled to reach CS maceration temperature (10 °C) at this point. Thus the end of CS for 1 day is shown at 39 h (time from start of cooling of different CS duration treatments in addition to actual CS maceration time at 10 °C) on the time line and subsequently 111, 183, and 255 h for 4, 7, and 10 days of CS. From the results shown in Figure 1, it can be seen that the color density increased with a longer cold maceration, although it did not persist beyond the CS period. The greatest color density at the end of the CS period was observed for the 10 days’ CS treatment at 4.14 AU (Table 2). These differences persisted for the first two days of active fermentation, but by day 4 few differences existed in color density for the treatments. By the end of fermentation there were no significant differences in color density values among the different CS duration wines.

**Table 2:** Color density and hue of wines made with different CS durations at the end of CS, the end of alcoholic fermentation (AF), and in bottled wines.

Absorbance Unit (AU)		Cold Soak Duration				
		0 Day	1 Day	4 Days	7 Days	10 Days
Color density (420 + 520 + 620 nm)	End of CS	−	0.97 ± 0.21c	2.51 ± 0.46b	3.41 ± 0.33ab	4.14 ± 0.15a
	End of AF	7.68 ± 0.09a	8.26 ± 0.56a	7.88 ± 0.22a	7.64 ± 0.06a	8.23 ± 0.37a
	Bottle	6.96 ± 0.12a	7.22 ± 0.42a	7.12 ± 0.24a	6.76 ± 0.17a	7.37 ± 0.06a
Hue (420/520 nm)	End of CS	−	0.86 ± 0.15a	0.42 ± 0.01ab	0.49 ± 0.01b	0.51 ± 0.02ab
	End of AF	0.47 ± 0.01b	0.49 ± 0.02ab	0.48 ± 0.02ab	0.54 ± 0.02a	0.51 ± 0.02ab
	Bottle	0.73 ± 0.01a	0.72 ± 0.01a	0.72 ± 0.02a	0.72 ± 0.01a	0.72 ± 0.01a

Notes: Means ± SD followed by same letter within the same row indicates no significant difference (*p* < 0.05, n = 3). (−): no analysis as there is no end of CS for non-CS treatment.

All finished wines were also analyzed five months after the end of treatment (three months’ bottle aging), and their color density and hue value are shown in Table 2. No significant differences were seen in either the color density or the hue among the different CS treatments in the bottled wine. The color density of the wines decreased by approximately 11% during the five-month post-treatment period, with a simultaneous increase of approximately 30% in hue. The decrease in color density and increase in hue value during aging are due to the loss of free anthocyanins as a result of polymerization and other modification reactions with other compounds in red wine to form polymeric pigments as well as degradation reactions [15,16,17]. The concentration of anthocyanins in red wines changes significantly during the first year of storage. Potential degradation reactions include glycoside hydrolysis or breakdown of the carbon chain of the chalcone molecule as a result of a shift in the equilibrium towards the colorless chalcone form [18].

### 2.3. Phenolic Composition of the Must and Wines

**Figure 2:**
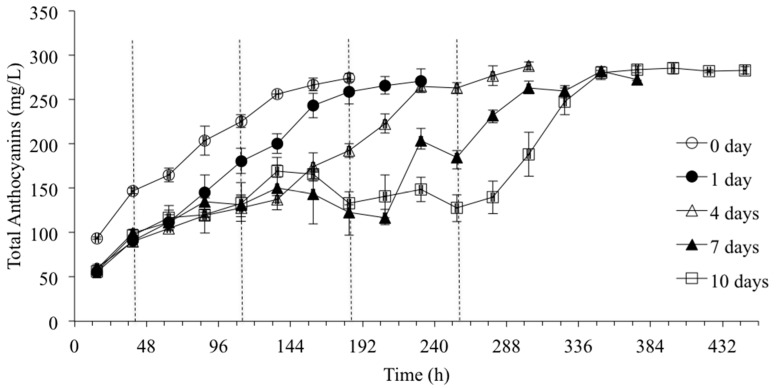
Total anthocyanin concentration in treatments with different CS durations during CS period and active fermentation, as determined by RP-HPLC (n = 3). The end of CS for 1, 4, 7, and 10 days of CS treatment are marked by a dashed line at 39, 111, 183, and 255 h, respectively.

Monomeric phenol concentrations were determined by RP-HPLC and tannin concentration was estimated by UV-vis using the Skogerson–Boulton model. Phenolic extraction profiles in the must and wine for different CS durations during CS, active fermentation, and in the finished wine after three months of bottle aging were determined. The extraction profiles of both hydroxycinnamates (caffeic acid, caftaric acid, coutaric acid, *p*-coumaric acid) and flavonols (quercetin, quercetin-glycosides) were similar to the extraction profile shown for total anthocyanins in Figure 2 (data not shown). Hydroxycinnamate extraction increased with CS duration but by the end of primary fermentation there were no significant differences between the different CS treatments. Although the extraction profile of the flavonols (quercetin and quercetin-glycosides) were similar to the hydroxycinnamates, a lower percentage was extracted during CS durations compared to during active fermentation due to lower solubility of the flavonols in the aqueous must. Higher temperature and increasing ethanol content during fermentation increased extraction and solubility [1], resulting in similar concentrations in the final wines made with different CS durations. Thus CS duration had no significant impact on the hydroxycinnamate and flavonol content of the finished wine (Table 3).

The extraction profiles of total anthocyanins during different CS durations and primary fermentation are shown in Figure 2. The evolution of extracted anthocyanins showed an almost constant increase during the CS period for all treatments. However, for the 7- and 10-day CStreatments a maximum was reached after five days of CS, followed by a decrease during the remainder of the CS period. According to Singleton and Trousdale [19], this decrease is due to the fact that during the maceration period, parallel to the extraction, the anthocyanins are slowly reacting with other compounds. Another possibility is readsorption of the extracted anthocyanins on to the grape cell walls, similar to their adsorption on to yeast cell walls during fermentation [20]. The significantly higher anthocyanin concentration for the non-CS treatment compared to the CS treatments at the first sampling point is due to faster extraction of anthocyanins at higher temperatures (25 °C *vs* 10 °C), as discussed previously in Section 2.2. The 1-, 4-, 7-, and 10-day CS treatments showed significantly higher anthocyanin concentrations at the start of fermentation (sample point after dashed lines) when compared to the control (first sample point) (Figure 2). Extraction of anthocyanins increased during fermentation due to increased fermentation temperature and ethanol generation [1]. The musts with longer CS duration, especially the 10-day CS treatment, also showed greater anthocyanin extraction during active fermentation, which is in agreement with a study that compared no CS with eight and 12 days of cold maceration [13]. The 10-day CS treatment showed greater rates of extraction during the first four days of fermentation (Figure 2). Faster extraction rates with longer CS periods could be due to increased permeability of the cell membranes as a result of longer contact time, which only becomes apparent during fermentation due to solubility limitations in the 10 °C aqueous must. However, at the end of fermentation all the treatments had similar concentrations of extracted anthocyanins, which persisted with bottle aging (Table 3). There were, however, significant differences in acylated anthocyanins. Both peonidin-3-glucoside-acetate and malvidin-3-glucoside-acetate concentrations were significantly lower in the finished wines made with longer CS duration (seven and 10 days) compared with the control. It has been shown that the profile of anthocyanin derivatives can be influenced by adsorption of anthocyanins onto yeast cell walls. The acylated anthocyanins are more strongly adsorbed onto yeast cell walls than non-acylated anthocyanins and the same may potentially be true for grape cell walls [20].

**Figure 3:**
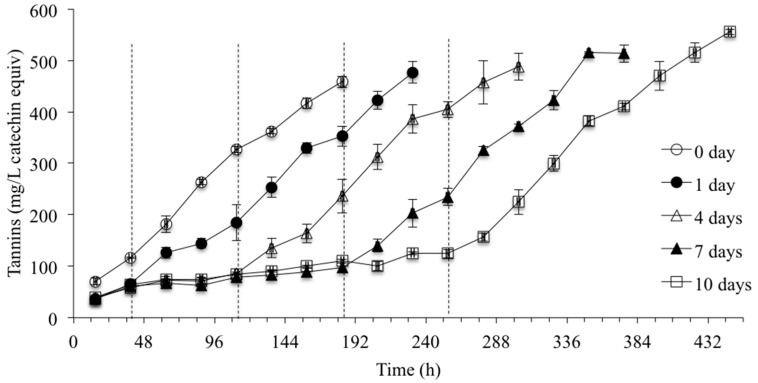
Tannin concentrations in treatments with different CS durations during CS period and active fermentation, as determined by the Skogerson–Boulton model (n = 3). The end of CS for 1, 4, 7, and 10 days of CS treatments are marked as a dash line at 39, 111, 183, and 255 h, respectively.

The amount of tannin extracted during CS and primary fermentation for different CS durations is shown in Figure 3. Increased amounts of tannin were extracted in the treatments with increasing CS duration, although there were no significant differences in total tannin concentration among the 1- to 10-day CS treatments at the end of CS. This is due to slow extraction into the aqueous medium, potentially reaching a temporary saturation point at 10 °C. Other related research in our laboratory supports this claim. With the onset of fermentation and the simultaneous increase in both fermentation temperature and ethanol content, extraction of tannins from both the grape skins and seeds continues. We hypothesize that the extended maceration time in the longer CS duration treatments resulted in the increased permeability of the cell walls, resulting in increased extraction of phenolics in these treatments when solubility of the compounds improved due to increased temperature and ethanol content [1,21,22]. The extraction profiles shown for tannin were also true for gallic acid (benzoic acid mainly present in the seeds and released as hydrolysis product) and the monomeric flavan-3-ols ((+)-catechin and (−)-epicatechin) monitored, with greater amounts extracted with increasing CS duration during CS and active fermentation. Differences in phenol concentrations (gallic acid, (+)-catechin, (−)-epicatechin, and tannin) at the end of fermentation persisted among the different CS duration wines (0 to 10 days) and were still present five months post-treatment (Table 3). The 4-, 7-, and 10-day CS treatments resulted in wines with significantly higher concentrations of gallic acid and monomeric flavan-3-ols when compared to the control wine, although there were no significant differences between the 4- to 10-day CS wines for monomeric flavan-3-ols.

**Table 3:** Concentration of phenolic compounds (mg/L ± SD) in finished wines made with different CS durations.

Compound	Cold Soak Duration					
	0 Day	1 Day	4 Days	7 Days	10 Days	
Gallic acid	27.40 ± 0.28d	28.43 ± 0.74cd	30.55 ± 2.15bc	32.54 ± 0.94ab	33.67 ± 1.14a	
(+)-Catechin	38.52 ± 1.05c	41.04 ± 2.06bc	43.98 ± 2.72ab	46.30 ± 2.07ab	47.27 ± 2.54a	
(−)-Epicatechin	17.65 ± 1.12c	19.10 ± 0.14bc	21.02 ± 0.93ab	22.33 ± 0.25a	22.74 ± 1.24a	
Caftaric acid	3.10 ± 0.31a	3.39 ± 0.55a	5.16 ± 1.42a	3.91 ± 2.03a	4.08 ± 1.25a	
Coutaric acid	1.47 ± 0.14a	1.42 ± 0.25a	2.15 ± 0.68a	1.70 ± 0.67a	1.73 ± 0.47a	
Caffeic acid	33.17 ± 0.75a	35.88 ± 1.04a	34.71 ± 2.77a	32.01 ± 2.91a	32.68 ± 1.36a	
*p*-Coumaric acid	10.77 ± 0.41a	11.79 ± 0.15a	11.42 ± 0.76a	10.37 ± 0.34a	10.24 ± 0.51a	
Quer-glycoside	12.11 ± 0.70a	11.38 ± 2.18a	10.99 ± 0.56a	9.90 ± 0.59a	10.51 ± 1.09a	
Quercetin	2.27 ± 0.55a	2.34 ± 0.28a	2.49 ± 0.45a	2.04 ± 0.10a	1.96 ± 0.42a	
Delph-3-glu	4.49 ± 0.13a	4.54 ± 0.64a	4.99 ± 0.09a	4.34 ± 0.11a	4.59 ± 0.27a	
Pet-3-glu	5.43 ± 0.26a	5.45 ± 0.59a	5.58 ± 0.28a	4.87 ± 0.17a	5.14 ± 0.26a	
Peo-3-glu	3.71 ± 0.37a	3.67 ± 0.29a	3.70 ± 0.05a	3.45 ± 0.05ab	3.00 ± 0.07b	
Mlv-3-glu	135.65 ± 8.40a	141.63 ± 2.84a	145.03 ± 8.39a	137.00 ± 0.93a	134.24 ± 6.09a	
Delph-3-glu-ac	1.63 ± 0.09a	1.68 ± 0.13a	1.66 ± 0.02a	1.48 ± 0.05a	1.46 ± 0.07a	
Pet-3-glu-ac	2.33 ± 0.15a	2.31 ± 0.17a	2.29 ± 0.10a	2.05 ± 0.06a	2.05 ± 0.10a	
Peo-3-glu-ac	1.56 ± 0.09a	1.56 ± 0.06ab	1.59 ± 0.02ab	1.52 ± 0.04bc	1.46 ± 0.05c	
Mlv-3-glu-ac	48.53 ± 4.40a	45.88 ± 2.84ab	44.26 ± 0.64ab	39.83 ± 1.64bc	37.71 ± 0.44c	
Pet-3-glu-cou	1.42 ± 0.05ab	1.43 ± 0.03a	1.44 ± 0.03a	1.37 ± 0.04ab	1.33 ± 0.01b	
Peo-3-glu-cou	1.56 ± 0.09a	1.56 ± 0.06a	1.59 ± 0.02a	1.52 ± 0.04a	1.46 ± 0.05a	
Mlv-3-glu-cou	11.22 ± 1.06a	10.92 ± 0.88a	11.23 ± 0.13a	10.25 ± 0.37a	9.82 ± 0.23a	
Total Anthocyanin	218.83 ± 14.70a	221.73 ± 8.53a	224.40 ± 9.53a	208.56 ± 1.60a	203.05 ± 5.49a	
Total tannin	436.31 ± 2.69c	451.64 ± 27.74bc	486.09 ± 32.52abc	496.78 ± 11.85ab	532.78 ± 15.54a	
Poly-pigment	17.21 ± 0.59a	17.51 ± 0.25a	17.06 ± 0.46a	16.37 ± 0.01a	16.96 ± 0.40a	

Notes: Means ± SD followed by same letter within the same row indicates no significant difference (*p* < 0.05, n = 3). Quer-glycoside, quercetin glycosides; Poly-pigment, Polymeric pigment; Delph-, Pet-, Peo-, and Mlv-3-glu; delphinidin-, petunidin-, peonidin-, and malvidin-3-glucoside, respectively. Delph-, Pet-, Peo-, and Mlv-3-glu-ac; delphinidin-, petunidin-, peonidin-, and malvidin-3-glucoside-acetate, respectively. Pet-, Peo-, and Mlv-3-glu-cou; petunidin-, peonidin-, and malvidin-3-glucoside-*p*-coumarate, respectively.

### 2.4. Proanthocyanidin Composition of Wines 

Proanthocyanidin composition of musts and wines were determined at the end of CS, the end of fermentation, and five months post-treatment by phloroglucinolysis (Table 4). There were no significant differences in the mean degree of polymerization (mDP), tannin concentration, and average molecular weight among the different CS treatments. However, there were some significant differences in the percentage galloylation of the different CS samples. In general, the percentage galloylation increased with increasing CS duration and increased from the end of CS to the end of fermentation for all treatments, followed by a decrease post-fermentation. There were also small but significant differences in percentage gallo units with generally a decrease with CS duration and an increase from the end of CS to the end of fermentation. The percentage gallo units and galloylation have been shown to be estimates of the skin (epigallocatechin subunit) and seed (epicatechin gallate subunit) tannin extracted into the wine, respectively. We can thus conclude from the results that seed tannin contribution increased in wines with longer CS duration. Tannins are extracted from skins and seeds during maceration, and it has been reported that skin tannins extract more readily, whereas extraction from seeds requires longer maceration and is accelerated by the presence of ethanol [22,23,24,25,26]. The longer CS contact time (seven to 10 days) also slightly increased tannin concentration at the end of fermentation and five months post-treatment, although it was not statistically significant. The differences in tannin concentration determined by the Skogerson–Boulton model and phloroglucinolysis are due to the fact that the Skogerson–Boulton model estimates protein precipitable tannin, which includes quantification of indirect polymerization products such as polymeric pigments, whereas phloroglucinolysis only quantifies grape skin and seed proanthocyanidins.

**Table 4:** Proanthocyanidin composition of wines made with different CS durations at the end of CS, the end of alcoholic fermentation (AF), and in finished wine, including the mean degree of polymerization (mDP), average tannin concentration, average molecular weight (MW), percentage galloylation, and percentage gallo units.

		Cold Soak Duration				
		0 Day	1 Day	4 Days	7 Days	10 Days
**mDP**	End of CS	−	6.86 ± 0.47a	6.62 ± 0.15a	6.88 ± 0.99a	5.95 ± 0.09a
	End of AF	10.97 ± 0.47a	11.36 ± 0.49a	10.92 ± 0.09a	10.82 ± 0.13a	11.00 ± 0.23a
	Bottle	10.86 ± 0.24a	10.94 ± 0.43a	10.37 ± 0.64a	10.17 ± 0.37a	10.60 ± 0.42a
**Tannin (mg/L)**	End of CS	−	43.98 ± 7.79a	47.95 ± 5.03a	41.49 ± 4.52a	43.19 ± 5.57a
	End of AF	623.66 ± 50.25a	546.71 ± 36.80a	569.09 ± 29.49a	628.07 ± 12.34a	675.76 ± 34.16a
	Bottle	518.17 ± 23.44a	499.15 ± 52.24a	513.54 ± 53.83a	543.35 ± 30.63a	612.25 ± 10.61a
**Average MW**	End of CS	−	2032.93 ± 137.62a	1958.04 ± 44.73a	2037.12 ± 300.03a	1751.95 ± 25.95a
	End of AF	3298.05 ± 143.90a	3414.46 ± 150.42a	3285.60 ± 23.19a	3261.52 ± 38.90a	3315.92 ± 68.56a
	Bottle	3235.54 ± 71.48a	3257.42 ± 128.91a	3085.02 ± 190.25a	3030.39 ± 111.92a	3161.16 ± 126.05a
**% Galloylation**	End of CS	−	3.01 ± 0.26a	2.51 ± 0.11a	2.78 ± 0.69a	2.35 ± 0.16a
	End of AF	4.86 ± 0.09b	4.73 ± 0.13b	5.05 ± 0.34ab	5.46 ± 0.19a	5.59 ± 0.06a
	Bottle	2.98 ± 0.07ab	2.84 ± 0.13b	2.78 ± 0.05b	3.18 ± 0.02ab	3.42 ± 0.10a
**% Gallo units**	End of CS	−	20.56 ± 2.27ab	21.73 ± 1.68a	19.06 ± 1.47ab	15.91 ± 2.34b
	End of AF	30.88 ± 0.57ab	31.27 ± 0.43a	30.08 ± 0.96abc	29.080.73bc	28.70 ± 0.33c
	Bottle	31.15 ± 0.80ab	32.14 ± 0.18a	31.11 ± 0.71ab	26.62 ± 1.27b	29.62 ± 0.35b

Notes: Means ± SD followed by same letter within the same row indicates no significant difference (*p* < 0.05, n = 3). (−) means no analysis as there is no end of CS for non-CS treatment.

**Figure 4:**
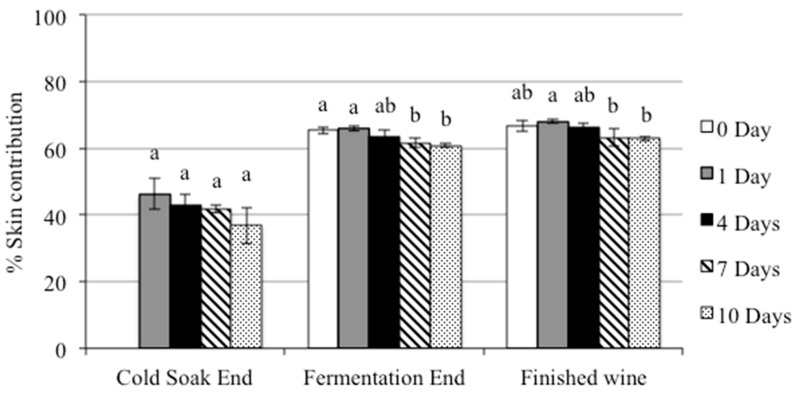
The percentage of skin tannin contribution with different CS durations at the end of CS, the end of fermentation and in finished wines. Means with different letters are significantly different (*p* < 0.05, n = 3).

The percentage of seed and skin tannin contribution in wine was also calculated using the method of Peyrot des Gachons and Kennedy [14]. The results are presented as the percentage skin tannin contribution in Figure 4. At the end of CS there was a slight decrease in skin tannin with longer CS durations, although the differences among treatments were not significant. At the end of fermentation, there were significant differences in the proportion of skin tannin between treatments of 0 and 1 day CS *versus* 7 and 10 days’ CS, confirming that the skin tannin proportion declined in relation to seed tannin with the longer CS durations. The percentage of seed tannin increased from 34.6% in the control to 39.3% in the 10-day CS treatment. Increases in the proportion of seed tannin may potentially affect the sensory profile of the wine. The differences in seed tannin contribution among treatments mostly persisted in the finished wine. It can be noted that CS duration had no significant effect on the total skin and seed tannin concentration but had a significant effect on the skin and seed tannin proportions. Similarly, Peyrot des Gachons and Kennedy [14] found increased seed tannin proportions at the beginning of fermentation when comparing four and 10 days of CS. However, they found no differences in skin and seed tannin proportions or final concentrations by the end of fermentation. This study showed that the seed tannin proportion and total protein precipitable tannin increased with the longer CS duration, and the 7- and 10-day CS treatments may potentially have increased bitterness and astringency (mouthfeel) in the final wines [27,28].

## 3. Experimental Section 

### 3.1. Harvest and Winemaking

Approximately 3000 kg of Cabernet Sauvignon grapes were harvested in 2013 from Lodi, California and received by the UC Davis Teaching and Research Winery (Davis, CA, USA) and immediately processed. The chemical characteristics of the grapes at harvest were 26 Brix, 4.4 g/L tartaric acid (TA), and pH of 3.7. Clusters (approximately 300 berries) were randomly selected and stored at −20 °C for further analysis prior to crushing. The grapes were destemmed and crushed using a Bucher Vaslin Delta E2 (Santa Rosa, CA, USA) directly into a Bucher Vaslin PMV must pump. The must was pumped into UC Davis/Cypress Semiconductor research fermentors. An addition of 15% potassium metabisulfite solution was performed for all fermenters, giving a final concentration of 60 mg/L sulfur dioxide (SO_2_).

To evaluate the effect of CS duration on grape skin and seed phenol extraction, 15 research-scale (75 L) fermentations were performed in jacketed, cylindrical, variable-capacity, stainless steel fermentor tanks with the fermentation conditions controlled by the Integrated Fermentation Control Systems (IFCS) units. All fermentations were cooled to 10 °C (overnight using jacket temperature and mixing) following addition of SO_2_ to prevent spontaneous fermentation. Experimental treatments were performed in triplicate with five CS durations investigated (0, 1, 4, 7, and 10 days). During CS pump-overs were performed twice per day with each pump-over volume being half of the must volume. At the end of the CS duration fermentations were heated to 25 °C prior to inoculation with *Saccharomyces cerevisiae* strain D254 (Lallemand Lalvin^®^). During fermentation two fermentor volumes were pumped over twice daily. All fermentations were performed at 25 °C with temperature maintained by means of the water jacket. Prior to inoculation, diammonium phosphate was added to increase the yeast assimilable nitrogen to 300 mg/L and tartaric acid was adjusted to 6 g/L. All treatments were sampled twice a day following pump-overs during the CS period and fermentation. The first sampling point was when CS maceration temperature was reached (10 °C) and fermentation started (conversion of sugar) in the no CS treatment, approximately 15 h after yeast inoculation. The total skin contact time of treatments after the end of alcoholic fermentation were 7.6, 9.6, 12.6, 15.6, and 18.6 days for 0, 1, 4, 7, and 10 days of CS treatment, respectively. Samples were centrifuged (Eppendorf Centrifuge 5810R, Eppendorf AG, Hamburg, Germany) at 3220 rpm for 15 min at 4 °C and subsequently stored at −20 °C until analysis.

Brix measurements were measured manually with an Anton Parr DMA35 density meter (Anton Parr, Ashland, VA, USA). Fermentations were pressed at 0 Brix using a prototype Cypress Semiconductor Corporation hydraulic basket press. Finished wines were inoculated with *Oenococcus oeni* (Chr. Hansen, Inc., Milwaukee, WI, USA) and all wines completed malolactic fermentation within four weeks.

After completion of malolactic fermentation, the free sulfur dioxide concentration was adjusted to 30 mg/L with the addition of a 15% potassium metabisulfite solution. All treatments were sterile filtered and bottled in 750 mL screw top bottles (Bordeaux style, green glass), purged with nitrogen gas prior to filling. The finished wines were stored at 14.4 °C in the Teaching and Research Winery at UC Davis until analysis three months after bottling.

The wine chemical compositions were determined at time of bottling for all treatments. The ethanol content was measured with an Alcolyzer (Anton Parr, Ashland, VA, USA). The pH was measured using an Orion 5-star pH meter (Thermo Scientific, MA, USA). The total acidity was measured automatically with the Mettler-Toledo DL50 titrator (Mettler-Toledo Inc., Columbus, OH, USA). The measurements of acetate, malate, and residual sugar were made using the Thermo Scientific Gallery automated analyzer (Thermo Scientific, MA, USA).

### 3.2. Grape Skin and Seed Tannin Extraction

Four sets of 20 berries were chosen at random from frozen clusters. Skins and seeds were separated from the berry mesocarp with a scalpel and were washed with deionized water, patted dry with paper towels, and weighed. The skins and seeds were extracted separately with 0.1 g of samples per 1 mL of 1:1 ethanol:water containing 0.1% v/v HCl and 0.1% w/v ascorbic acid. All samples were homogenized using an IKA ULTRA-TURRAX^®^T18 basic (IKA^®^ Works, Inc., NC, USA) and allowed to extract at 4 °C overnight. The samples were centrifuged at 3220 rpm for 15 min, after which the supernatant was collected. The homogenized samples were subsequently extracted with a 70:30 acetone:water solution containing 0.1% w/v ascorbic acid, which was added in the same ratio of sample to solvent as that of the ethanol solution and allowed to extract at 4 °C overnight. Samples were then centrifuged and the supernatants combined prior to concentration under reduced pressure at 35 °C followed by lyophilization.

### 3.3. Determination of Color and Adams–Harbertson Assay Correlation

Absorbance measurements were made using a Hewlett-Packard 8453 UV-vis spectrophotometer (Hewlett-Packard, Palo Alto, CA, USA) with 0.1 mm path length flow cell (Starna Cells, Atascadero, CA, USA). The frozen samples were thawed at room temperature, centrifuged, and absorption spectra were collected from 230–900 nm. Color density was calculated as the sum of absorbance at 420, 520, and 620 nm, and hue was calculated as the ratio between absorbance at 420 and 520 nm. The predicted Adams–Harbertson values for tannins with the coefficient of determination of (r^2^) 0.86 were generated for all samples using the Skogerson–Boulton model [29].

### 3.4. Reagents

Gallic acid monohydrate, (+)-catechin, (−)-epicatechin, caffeic acid, quercetin, quercetin-rhamnoside, *trans*-ferulic acid, and *p*-coumaric acid were purchased from Sigma Chemical Co. (St. Louis, MO, USA). Malvidin-3-*O*-glucoside chloride was purchased from Extrasynthese (Genay, France). Acetonitrile (Sigma Chemical Co., St. Louis, MO, USA) and formic acid (Fisher Scientific, Bridgewater, NJ, USA) were HPLC grade. HPLC-grade water was prepared in house to a final resistance of 18 MΩ and filtered through a 0.22 μm filter prior to use.

### 3.5. Reversed Phase HPLC (RP-HPLC) Analysis of Monomeric Phenols

Frozen wine samples were thawed, centrifuged, and filtered through 0.45 μm PTFE syringe-tip filters (Econo filter 25 mm, Agilent, Wilmington, DE, USA) prior to analysis. All wine samples were analyzed by RP-HPLC using an Agilent (Santa Clara, CA, USA) 1260 Infinity HPLC equipped with a binary pump, column compartment, and diode array detector. The column used was an Agilent Poroshell 120 SB-C18 (4.6 × 150 mm. 2.7 µm particle) maintained at 35 °C. The mobile phases used for the separation were solvent A (water with 5% v/v formic acid) and solvent B (10% v/v solvent A in acetonitrile). The mobile phase flow rate was set at 1.25 mL/min and 20 μL injection volumes were used for all samples. The gradient for the separation was 0–23 min, 5%–27% B; 23–24 min, 27%–95% B; 24–26 min, 95% B; 26–26.5 min, 95%–5% B; 26.5–32 min, 5% B. Eluting peaks were monitored at 280 (gallic acid, (+)-catechin, (−)-epicatechin, polymeric phenols), 320 (caftaric acid, caffeic acid, coutaric acid, *p*-coumaric acid), 370 (quercetin-3-galactoside, quercetin-3-glucuronide, quercetin-3-glucoside, quercetin), and 520 nm (anthocyanins, polymeric pigment). Compounds eluting from the HPLC were identified and quantified based on spectral and retention time comparisons to authentic standards. Phenolics were quantitated by external calibration, with calibration curves generated for gallic acid, (+)-catechin, (−)-epicatechin, caffeic acid, quercetin, quercetin-rhamnoside, *p*-coumaric acid, and malvidin-3-*O*-glucoside chloride. Chromatograms were integrated using Agilent^®^ CDS ChemStation software. Compounds were quantified as themselves if an authentic standard was available; otherwise they were quantified as follows: polymeric phenols as (+)-catechin equivalents; caftaric acid as caffeic acid equivalents; coutaric acid, as *p*-coumaric acid equivalents; quercetin-3-galactoside, quercetin-3-glucuronide, quercetin-3-glucoside as quercetin-3-rhamnoside equivalents; and anthocyanins and polymeric pigments as malvidin-3-*O*-glucoside chloride equivalents.

### 3.6. Isolation and Characterization of Proanthocyanidins 

Solid phase extraction (SPE) was performed to isolate tannins in triplicate for each sample using the method of Oberholster *et al.* [30]. The proanthocyanidins were purified using Toyopearl^®^ HW-40F size exclusion media. The columns were packed to a bed volume of 10 mL and equilibrated with 20 mL of 55:45 ethanol/water containing 0.05% *v*/*v* trifluoroacetic acid (TFA). Lyophilized seed and skin extracts were dissolved in 15% methanol solution at concentrations of 5 mg/mL and 10 mg/mL respectively. Reconstituted extracts were centrifuged at 3220 rpm for 15 min and loaded onto columns, with 1 mL for seed and 2 mL for skin extract solutions. Wine samples were thawed and centrifuged, and 2 mL were loaded onto the conditioned column. Sugars, protein, low molecular weight flavan-3-ols (monomers and dimers), and all other monomeric phenols were eluted with 40 mL of 55:45 ethanol:water containing 0.05% TFA. The proanthocyanidins were then eluted with 30 mL of 60:40 acetone:water containing 0.05% TFA. The proanthocyanidin fraction was concentrated under reduced pressure at 35 °C to remove all solvents and then dissolved in 0.5 mL of methanol. The concentrated proanthocyanidin samples were stored at −20 °C for a maximum of one month prior to analysis by phloroglucinolysis.

The isolated proanthocyanidins were analyzed using the phloroglucinolysis method optimized by Kennedy and Jones [31]. A phloroglucinol solution of 0.2 N HCl in MeOH containing 100 g/L phloroglucinol and 20 g/L ascorbic acid was prepared. The phloroglucinolysis reactions were performed in duplicate. Equal volume aliquots of the proanthocyanidin fraction and phloroglucinol solution were mixed and heated at 50 °C for 20 min. The reaction was quenched by the addition of five reaction volumes of 40 mM aqueous sodium acetate. Quenched digests were centrifuged for 5 min at 13,000 rpm (Eppendorf Centrifuge 5415D) and transferred into an HPLC vials. Due to the instability of the cleavage products, sample vials were held for a maximum of 12 h. An Agilent^®^ Infinity series 1260 HPLC was used for all phloroglucinolysis analyses. Phloroglucinolysis reaction products were analyzed using RP-HPLC with an Agilent Poroshell 120 SB-C18 (4.6 × 150 mm. 2.7 µm particle) HPLC column utilizing a binary gradient system of water with 0.1% formic acid (mobile phase A) and acetonitrile with 0.1% formic acid (mobile phase B). The mobile phase flow rate was set at 2.0 mL/min, 10 μL injection volumes were used for seed samples, and 20 μL injection volumes were used for skin and wine samples. The gradient for the separation was 0–2.96 min, 3% B; 2.96–10.30 min, 3%–16% B; 10.30–10.40 min, 16%–20% B; 10.40–12.10 min, 20% B; 12.10–13.0 min, 20%–80% B; 13.0–14.34 min, 80% B; 14.34–15.34 min, 80%–3% B; 15.34–20.0 min, 3% B. The column temperature was maintained at 35 °C and the eluting peaks were monitored at 280 nm. Quantitation of reaction products was performed using an external calibration generated with (+)-catechin using their response factor relative to catechin and with molar extinction coefficients corrected using values determined by Kennedy and Jones [31]. The chromatographs were integrated using Agilent^®^ CDS ChemStation software. Tannin concentration, mean degree of polymerization (mDP), percentage galloylation, and percentage gallo units were determined for each sample.

The percentage of seed and skin tannin contribution in wine samples was also calculated by comparing the proportional extension subunit composition in wine relative to the proportional extension subunit in the corresponding grape [14].

### 3.7. Statistical Analysis

Statistical analysis was performed using R (ver. 2.15.1). Fisher’s least significant differences (LSD) were used to discriminate the means between all fermentation treatments using the function found in the Agricolae package, which was built under R version 2.15.1.

## 4. Conclusions 

It can be concluded that Cabernet Sauvignon wine made with a CS duration of 4 to 10 days at 10 °C had greater extraction of phenolic compounds (gallic acid, (+)-catechin, (−)-epicatechin, and tannin) when compared to the control that did not experience CS. Not all phenolic compounds increased with longer CS duration as there was no significant effect on hydroxycinamate and flavonol extractions during fermentation and these results also persisted in the final wine. Although anthocyanin extraction and color density increased with longer periods of CS, there were no significant differences due to CS duration by the end of primary fermentation. Furthermore, 7 and 10 days of CS resulted in wines with higher seed tannin proportions and total tannin when compared to the non-CS wines and potentially enhanced bitterness and astringency in the wines.
